# The Office of Coroner Abolished

**Published:** 1902-05

**Authors:** 


### The Office of Coroner Abolished.

FOLLOWING the plan in vogue in Massachusetts for many years the last legislature, at the instance of Senator George A. Davis, of this county, passed a law discontinuing the time-honored office of coroner in the County of Erie. This is in accordance with the spirit of the age and coincides with the action taken in the Medical Society of the State of New York some years ago, when it endorsed and urged a bill to abolish the office of coroner throughout the state.

The coroner is one of the oldest officers recognised by law; he was an officer of the crown and commanded the highest respect as a public functionary, representing, as he did, the authority of the king, wherever he went in discharge of his duties. The traditions of "crowners' quest law" have furnished theme for comment in classic literature and everybody is familiar with the clownish grave-diggers exposition of it in Hamlet. In this county the office has existed from the first, it being an heir-loom, so to speak, from colonial times, handed down in turn from the mother country.

The change, therefore, which is taking place is a radical one, but it is believed by those most familiar with the new plan that it is much better than the old one, and that the ends of justice will be served more adequately by it.

The new law substitutes the office of medical examiner for that of coroner,—an administrative office for a semi-judicial one. The medical examiner will conduct all inquiries relating to the cause of sudden death, including both medical and legal aspects of the case, and report his findings to the District Attorney, filing with him papers and evidence in criminal cases. He is given an assistant who is empowered to act in his absence, disability, or in his stead, if for any reason he is inaccessible. A secretary also is provided, who has immediate charge of the office and its records.

Dr. Earl G. Danser, who was elected coroner last fall by a very large majority, has been appointed to the office of medical examiner, and will inaugurate the new service, with the best wishes of his professional colleagues, who will assuredly give him their unstinted support to make the service dignified, scientific, and creditable in every way. Dr. Danser's selection by the board of supervisors, was the logical solution of the difficulties growing out of the change. He was elected coroner by many thousand votes, his office was abolished during his term, and he is capable and honest. That was all there was of it and the board took this very sensible view. Dr. J. D. Howland, who was elected assistant medical examiner, we have every reason to believe will prove an excellent officer. The JOURNAL extends its best wishes to the new officers, and its best thanks to Senator Davis for his successful efforts in securing the passage of the law that has in it so much of promise for improvement.

